# Plasma IL-1Ra: linking hyperapoB to risk factors for type 2 diabetes independent of obesity in humans

**DOI:** 10.1038/nutd.2015.30

**Published:** 2015-09-28

**Authors:** S Bissonnette, N Saint-Pierre, V Lamantia, Y Cyr, H Wassef, M Faraj

**Affiliations:** 1 Faculty of Medicine, Université de Montréal, Montréal, Québec, Canada; 2 Département de Nutrition, Institut de Recherches Cliniques de Montréal (IRCM), Montréal, Québec, Canada; 3 Montreal Diabetes Research Center (MDRC), Montréal, Québec, Canada

## Abstract

**Background/Objective:**

Plasma apoB predicts the incidence of type 2 diabetes (T2D); however, the link between apoB-linpoproteins and risks for T2D remain unclear. Insulin resistance (IR) and compensatory hyperinsulinemia characterize prediabetes, and the involvement of an activated interleukin-1 (IL-1) family, mainly IL-1β and its receptor antagonist (IL-Ra), is well documented. ApoB-lipoproteins were reported to promote IL-1β secretion in immune cells; however, *in vivo* evidence is lacking. We hypothesized that obese subjects with hyperapoB have an activated IL-1 system that explains hyperinsulinemia and IR in these subjects.

**Subjects/Methods:**

We examined 81 well-characterized normoglycemic men and postmenopausal women (⩾27 kg m^−2^, 45–74 years, non-smokers, sedentary, free of chronic disease). Insulin secretion and sensitivity were measured by the gold-standard Botnia clamp, which is a combination of a 1-h intravenous glucose tolerance test (IVGTT) followed by 3-h hyperinsulinemic euglycemic clamp.

**Results:**

Plasma IL-1β was near detection limit (0.071–0.216 pg ml^−1^), while IL-1Ra accumulated at 1000-folds higher (77–1068 pg ml^−1^). Plasma apoB (0.34–1.80 g l^−1^) associated significantly with hypersinsulinemia (total_IVGTT_: C-peptide *r*=0.27, insulin *r*=0.22), IR (M/I=−0.29) and plasma IL-1Ra (*r*=0.26) but not with IL-1β. Plasma IL-1Ra associated with plasma IL-1β (*r*=0.40), and more strongly with hyperinsulinemia and IR than apoB, while the association of plasma IL-1β was limited to second phase and total insulin secretion (*r*=0.23). Adjusting the association of plasma apoB to hyperinsulinemia and IR for IL-1Ra eliminated these associations. Furthermore, despite equivalent body composition, subjects with hyperapoB (⩾80th percentile, 1.14 g l^−1^) had higher C-peptide secretion and lower insulin sensitivity than those with low plasma apoB (⩽20th percentile, 0.78 g l^−1^). Adjustment for plasma IL-1 Ra eliminated all group differences.

**Conclusion:**

Plasma apoB is associated with hyperinsulinemia and IR in normoglycemic obese subjects, which is eliminated upon adjustment for plasma IL-1Ra. This may implicate the IL-1 family in elevated risks for T2D in obese subjects with hyperapoB.

## Introduction

Normal plasma glucose is maintained by insulin sensitivity that is balanced by insulin secretion,^1, 2^ which increases in prediabetic states as a compensatory mechanism for increased insulin resistance (IR).^2, 3^ In time, this is believed to promote β-cells exhaustion, reduction in insulin secretion and type 2 diabetes (T2D).^4^ HyperapoB, or elevated concentrations of atherogenic ApoB-lipoproteins, is among the most common dyslipoproteinemia in subjects with IR and T2D, increasing morbidity and mortality in this population.^5^ However, accumulating evidence from our lab and others suggest that hyperapoB may precede and promote the development of T2D. ApoB-lipoproteins were shown to reduce white adipose tissue function^6, 7, 8, 9, 10, 11, 12^ and promote abnormalities in insulin action and secretion in muscle^9, 13^ and pancreatic cells.^6, 7, 8, 9, 10, 11, 12^ Accumulating epidemiological evidence confirmed that plasma apoB predicted T2D 3–10 years before its onset in Turkish (*N*=2248 women only),^14^ Canadian aboriginals (*N*=492),^15^ Finnish (*N*=12 804)^16^ and Korean (*N*=48 394)^17^ populations, independent of traditional risk factors such as age,^14, 15, 16^ sex,^15, 16^ smoking status,^16^ central adiposity,^15^ hypertension,^15^ and fasting plasma glucose,^15, 16, 17^ lipids^16, 17^ and glycated hemoglobin.^17^ Taken together, this suggests that subjects with hyperapoB may have increased risk for T2D before normal fasting glucose is affected. However, the clinical evidence is limited.

Although the etiology of T2D is mutifaceted, chronic subclinical inflammation particularly involving the interleukin-1β (IL-1β) is believed to have a major role in this process. Interleukin-1β is a master regulator of inflammation and has long been implicated in the pathology of T1D and more recently T2D.^18, 19^ IL-1β promotes pancreatic β-cell destruction as well as IR in insulin-sensitive tissue, such as muscle and white adipose tissue.^20, 21^ IL-1 receptor antagonist (IL-1Ra) is produced in response to IL-1β as a compensatory mechanism to block IL-1β actions by competitive binding to IL-1R without intracellular signalling.^22^ An increased local β-cell production of IL-1β relative to IL-1Ra is believed to promote β-cell toxicity, apoptosis and T2D.^20, 21, 23^ In circulation, however, plasma IL-1β is near detection limit in healthy subjects and the physiologically relevance of circulating, in face of locally produced, IL-1β is unknown.^24^ On the other hand, IL-1Ra accumulates in the plasma, and elevated plasma IL-1Ra were reported in subjects with obesity,^23^ impaired glucose tolerance^25^ and metabolic syndrome.^26^ Gradual increases in IL-1Ra concentrations were also reported in subjects ranging from prediabetic to diabetic states.^27^ Similar to hyperapoB, plasma IL-1Ra predicts the onset of T2D by 10 years independent of traditional risk factors, such as age and body mass index (BMI).^28, 29^ Elevated plasma IL-1Ra in prediabetic states are believed to be the body's attempt to offset the detrimental effects of increased IL-1β production and to preserve insulin secretion and sensitivity, efforts that eventually fail,^24, 29^ as T2D patients have low plasma IL-1Ra.^30^

IL-1β is the end product of the activation of the NLRP3 inflammasome (nucleotide-binding leucine-rich repeat-containing pyrin receptor 3), an innate immunity-related complex of intracellular proteins implicated in the recognition of obesity-associated metabolic signals in macrophage and pancreatic cells.^19, 20^ ApoB-lipoproteins, very low-density lipoprotein (LDL)^31^ and oxidized LDL^32^ were reported to activate the NLRP3 inflammasome leading to IL-1β secretion from monocytes and macrophages. This suggests that subjects with hyperapoB may also have an activated inflammasome leading to elevated production of IL-1β and IL-1Ra and explaining increased risks for T2D in this population. However, clinical evidence supporting this is lacking.

Here we tested the hypotheses that elevated plasma apoB is associated with (1) activated IL-1 family *in vivo* (that is, elevated plasma IL-1β and IL-1Ra) and (2) hyperinsulinemia and IR in an IL-1 family-dependent manner. To explore our hypotheses, we employed a gold-standard technique, the Botnia clamp, to measure glucose-induced insulin secretion and insulin sensitivity in 81 well-characterized overweight and obese non-diabetic men and postmenopausal women.

## MATERIALS AND METHODS

### Study population

Metabolic studies measuring insulin sensitivity and secretion *in vivo* were conducted between 2010 and 2014 at the Clinical Research Institute of Montreal (IRCM) with the following inclusion criteria as previously reported:^33^ BMI>kg m^−^^2^, age=45–74 years, confirmed menopausal status (follicle-stimulating hormone ⩾30 U l^−1^or >1 year without menses), non-smoker, sedentary (<2 h of structured exercise week^−1^), and low alcohol consumption (<2 alcoholic drinks day^−1^). The exclusion criteria were: (1) history of crdiovascular disease and hypertension requiring medication, (2) diabetes (or fasting glucose >7 mmol l^−1^), (3) cancer (within the past 3 years), (4) untreated thyroid disease, kidney disease (or creatinine >100 μmol l^−1^) or hepatic disease (or aspartate aminotransferase/alanine transminase >3 times normal limit), (5) claustrophobia, (6) anemia (Hb<120 g l^−1^) and blood coagulation problems, (7) current or past 3 months' use of drugs affecting metabolism (hormone-replacement therapy except thyroid hormone at a stable dose, systemic corticosteroids, antipsychotic/psycho-active drugs, anticoagulant, weight loss and adrenergic agonist), (8) known substance abuse, (9) exceeding the annual allowed radiation dose exposure, and (10) all other medical or psychological conditions deemed inappropriate according to the physician.

Out of the 110 subjects recruited, 82 were eligible and were included in this study (49 women and 33 men). One woman who was included in another genetic study at IRCM had a familial mutation that affects the metabolism of apoB-lipoproteins and was thus excluded from this analysis. All subjects signed an informed consent prior to initiation of the study, which was approved by the Ethics Board of Montreal Clinical Research Institute (IRCM).

### Anthropometry and metabolic measures

After a 4-week weight-stabilization period (that is, ±2 kg), body composition was measured by dual-energy X-ray absorptiometry (intelligent or iDXA, GE Healthcare, Little Chalfont, UK), which measures total body fat as well as android or central fat mass (starting above the pelvis), and gynoid fat mass (comprising the hips and thighs). Plasma lipids, apoA-1 and apoB were measured by an automated analyzer COBAS 400 (Roche Diagnostic, Basel, Switzerland), glucose by automated analyzer (YSI Incorporated, Yellow Springs, OH, USA), insulin by human insulin Radioimmunoassay Kit (Millipore Corporation, Billerica, MA, USA) and LDL diameter by an automated electrophoresis family (Lipoprint, Food and Drug Administration approved, Quantimetrix, Redondo Beach, CA, USA).^7, 33, 34^ Plasma IL-1β and IL-1Ra were measured by commercial high-sensitivity enzyme-linked immunosorbent assay (hsELISA) kits (R&D system, Minneapolis, MN, USA). The lower detection limits for plasma IL-1β of the kit was 0.057 pg ml^−1^ while that for IL-1Ra was 6.3 pg ml^−1^.

### Insulin sensitivity and secretion

**Figure 1:**
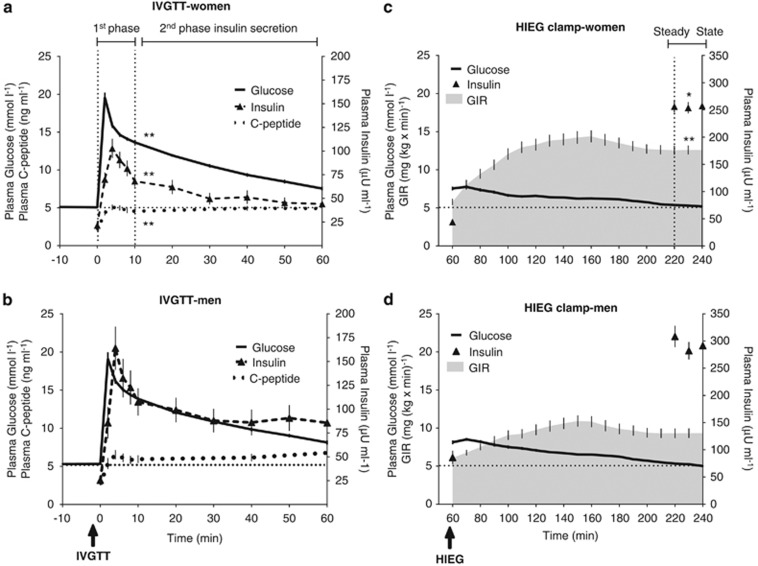
Plasma glucose, insulin and C-peptide and glucose infusion rates (GIR) during the IVGTT in women (**a**) and men (**b**) and the hyperinsulinemia euglycemia clamp (HIEG) in women (**c**) and men (**d**), the two-component test of the Botnia clamp. Sex difference at *P⩽0.05 and **P⩽0.01.

Concomitant assessment of insulin sensitivity and secretion was conducted using a modified Botnia clamp. In brief, subjects underwent a 1-h intravenous glucose tolerance test (IVGTT) using a bolus infusion of 20% dextrose (0.3 g glucose per kg body weight).^33, 35^ This was followed by a 3-h hyperinsulinemic euglycemic clamp, during which plasma insulin was elevated to a plateau concentration using a primed exogenous constant insulin infusion (75 mU m^−2^ min^−1^), while plasma glucose was maintained within fasting range (4.5–5.5 mm) by 20% dextrose infusion as previously published.^6, 33, 36, 37, 38^ First phase, second phase and total IS during the IVGTT were assessed as the area under the curve of plasma insulin during the first 10 min (AUC_10 min_), last 50 min (AUC_50 min_) or the total 60 min (AUC_60 min_) of the IVGTT, respectively. Total C-peptide secretion during the IVGTT was assessed as the AUC of the plasma C-peptide during the total 60 min of the IVGTT. Insulin sensitivity during the steady state of the clamp (last 30 min) was assessed as glucose infusion rate (GIR)/steady-state plasma insulin (M/I).^6, 33, 36, 37, 38^ Fasting indices of insulin sensitivity (QUICKI) was calculated as (1/(log(fasting insulin μU ml^−1^)+log(fasting glucose mg dl^−1^))) as published.^37^ Disposition index during the Botnia clamp was calculated as insulin sensitivity (expressed as M/I) multiplied by first phase or total insulin secretion during the IVGTT.^39^ All subjects were placed on a high carbohydrate diet (300 g day^−1^ for men and 225 g day^−1^ for women) for the 3 days preceding the Botnia clamp to maximize glycogen stores. Given that, to our knowledge, this is the first time that Botnia Clamp data are presented in overweight and obese subjects, the full clamp data are presented in Figure 1.

### Statistics

Data are presented as mean±s.e.m. Sex differences in Table 1 were analyzed by two-tailed *t*-test. Pearson correlation was used to examine the association between the variables in the whole group of 81 subjects. Slope analysis revealed no sex differences in the direction of associations for men and women, thus data remained pooled in all analysis. Data were log transformed when equal variance of residual values around the correlation line failed. All analysis using total, first-phase and second-phase insulin secretion, total C-peptide secretion, M/I and plasma IL-1β and IL-1Ra used the Log_10_ of these variables. Partial regression analysis was used for correction for obesity and body composition indices, plasma IL-1Ra and sex (as factor entered as 0 vs 1). Group differences in subjects with low and high plasma apoB were analyzed by General Linear Model univariate analysis with correction for obesity and body composition indices, plasma IL-1Ra and sex. Statistical analyses and slope analysis were performed using SPSS V22 (IBM, Armonk, NY, USA) and GraphPad Prism (version 6.03, Graphpad Software, La Jolla, CA, USA), and significance was set at *P*<0.05.

## Results

### Insulin sensitivity and secretion

Fasting baseline characteristics and indices of insulin sensitivity and secretion during the Botnia clamp of the 48 women and 33 men subjects are presented in Table 1. Plasma IL-1β was close to the detection limit of the hsELISA kit (0.057 pg ml^−1^) ranging from 0.071 to 0.216 pg ml^−1^, while plasma IL-1Ra ranged from 77 to 1068 pg ml^−1^, >10-folds higher than the detection limit of the hsELISA kit (6.3 pg ml^−1^) and 1000-folds higher than plasma IL-1β. Sex differences existed in adiposity, fat distribution and plasma lipids but not in plasma apoB, IL-1β or IL-1Ra.

**Figure 2:**
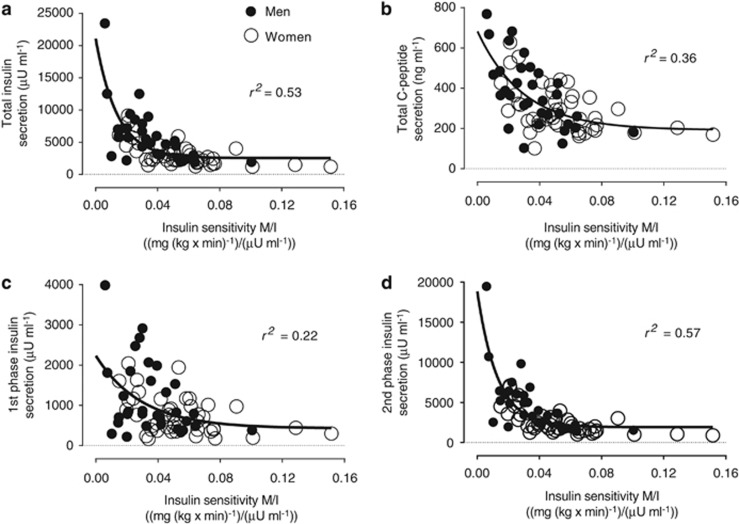
Hyperbolic relation (one phase decay) of insulin sensitivity with total insulin secretion (**a**), total C-peptide secretion (**b**), first-phase insulin secretion (**c**) and second-phase insulin secretion (**d**) in women (open circles) and men (closed circles).

A hyperbolic relationship existed between insulin sensitivity and secretion when measured using two separate tests, and whether insulin (Figure 2a) or C-peptide (Figure 2b) were evaluated, and was mainly driven by second-phase insulin secretion (Figure 2D, ~78% of total). This is in concordance with that reported using a single test, whether by frequently sampled IVGTT^39^ or oral GTT.^2^ Despite comparable plasma glucose, HbA_1_C and disposition index (first phase or total insulin secretion), men had higher indices of insulin secretion (fasting C-peptide, first phase, second phase and total insulin and C-peptide secretions) while women has higher indices of insulin sensitivity (QUICKI, GIR_clamp_ and M/I_clamp_) (Table 1 and Figures 1 and 2). As anticipated, obesity indices, particularly android, associated positively with hyperinsulinemia, IR and IL-1Ra (but not IL-1β Supplementary Table S1). Correcting for android/gynoid fat ratio, waist/hip circumference ratio or lean body mass eliminated all these sex differences, while correcting for any other index (BMI, total, gynoid fat or android fat mass or waist circumference) had no effect.

### Relation of plasma apoB to plasma IL-1β and IL-1Ra, hyperinsulinemia and IR

**Figure 3:**
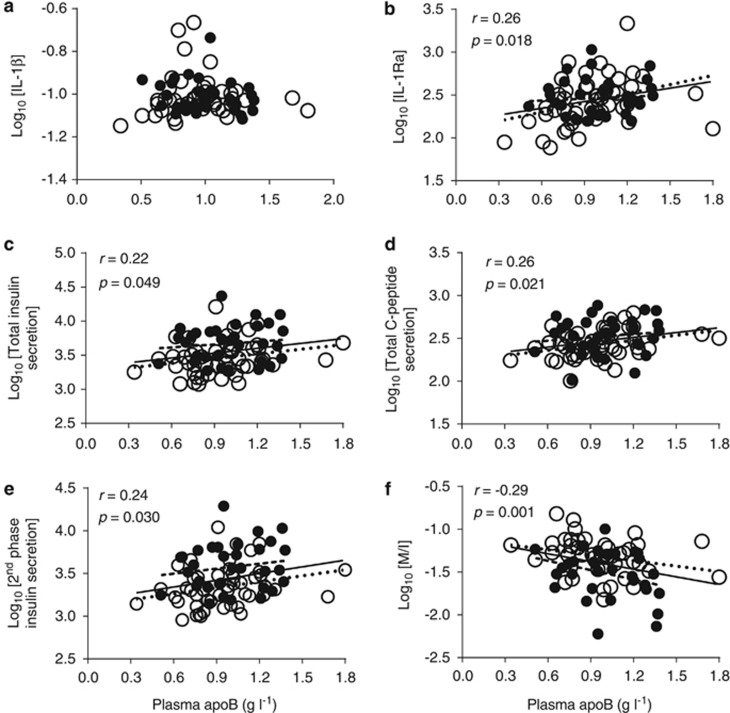
Correlation of plasma apoB with plasma IL-1β (**a**), IL-1Ra (**b**), total insulin secretion (**c**), total C-peptide secretion (**d**), second-phase insulin secretion (**e**) and insulin sensitivity (**f**) in women (open circles, dotted slope line) and men (solid circles, dashed slope line). Solid slope line represents pooled men and women data.

As hypothesized, plasma apoB correlated with plasma IL-1Ra (Figure 3b) though not with IL-1β (Figure 3a). Moreover, plasma apoB correlated positively with total insulin (Figure 3c) and C-peptide (Figure 3d) secretions, mainly second-phase insulin secretion (Figure 3e) but not first phase. It also correlated negatively with insulin sensitivity measured during the hyperinsulinemia clamp (Figure 3f), as was previously reported in similar populations,^6, 33^ but not with that at fasting (Supplementary Table S1). Notably, there was no association between plasma apoB with any index of body composition and with no sex differences (Supplementary Figure S1). Nevertheless, to verify that the association of plasma apoB to hyperinsulinemia, IR and plasma IL-1Ra was independent of adiposity, we used a partial correlation analysis. Adjusting for BMI, total, gynoid or android fat mass, lean body mass, waist or waist/hip ratio did not eliminate any of the associations in Figure 3, while adjusting for android/gynoid fat mass only eliminated that with total insulin secretion. This suggests that the relation of plasma apoB to plasma IL-1Ra, hyperinsulinemia and IR is, in general, independent of body composition in this population.

Finally, given the hyperbolic relation between insulin sensitivity and secretion, we tested whether the association of apoB to glucose-induced insulin secretion was dependent on its association with IR. Adjusting for insulin sensitivity (Log_10_M/I) increased the association of plasma apoB to Log_10_ second phase and total insulin secretion (*r*=0.894 and *r*=0.889, respectively, *P*<0.001). C-peptide is, however, a better index of insulin secretion given its longer half-life than insulin (20–30 vs 3–5 min),^40^ and adjusting for insulin sensitivity (Log_10_M/I) eliminated the association of plasma apoB with Log_10_ C-peptide. This suggests that the association of apoB with insulin secretion is dependent on its association with IR.

### Relation of plasma IL-1 family to hyperinsulinemia and IR

**Figure 4:**
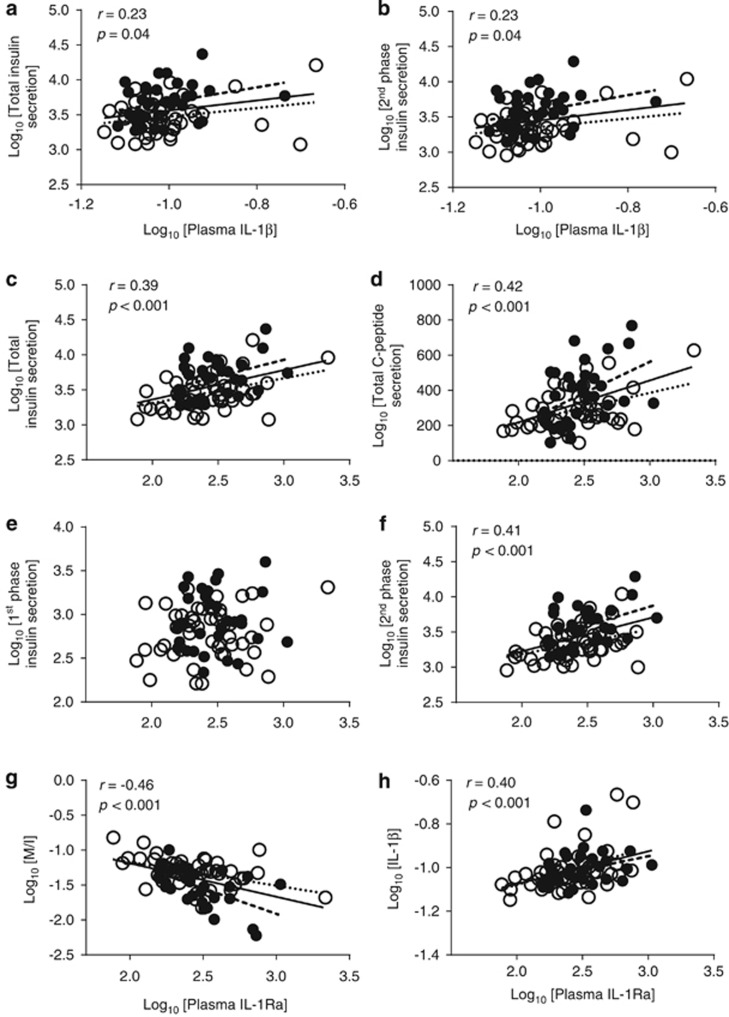
Correlation of plasma IL-1β with total (**a**) and second-phase insulin secretion (**b**) and IL-1Ra with total insulin secretion (**c**), total C-peptide (**d**), first-phase insulin secretion (**e**), second-phase insulin secretion (**f**), insulin sensitivity (**g**) and plasma IL-1β (**h**), in women (open circles, dotted slope line) and men (solid circles, dashed slope line). Solid slope line represents pooled men and women data.

To test whether the relation of plasma apoB to hyperinsulinemia and IR was dependent on the IL-1 family, we first examined whether plasma IL-1β and its receptor antagonist were related to hyperinsulinemia and IR during the Botnia clamp in an obese population, which to our knowledge has never been reported. Plasma IL-1β correlated with total and second-phase insulin secretion (Figures 4a and b) but not with insulin sensitivity (fasting or clamp indices, *P*>0.05). On the other hand, plasma IL-1Ra correlated with glucose-stimulated insulin and C-peptide secretions (Figures 4c, d and f) and with IR (Figure 4g). Moreover, there was a positive correlation between plasma IL-1Ra and IL-1β (Figure 4h), which supports that increased IL-1Ra is secreted, at least in part, in response to increased IL-1β tissue production. It also supports the use of plasma IL-1Ra as an index for systemic IL-1β, given that circulating IL-1β is near detection limits in healthy subjects.

White adipose tissue is a source of IL-1Ra, and plasma IL-1Ra is elevated in obesity,^41, 42^ which is also demonstrated in this study (Supplementary Table S1). Adjusting the associations of plasma IL-1Ra with insulin sensitivity and secretion for total or android fat only eliminated its association with total C-peptide secretion, while adjusting for android fat also eliminated its association with M/I and total insulin secretion. Adjusting for any other obesity index (BMI, gynoid fat mass, android/gynoid fat mass ratio, waist circumference or waist/hip ratio) had no effect. Finally, when insulin sensitivity (Log_10_ M/I) was adjusted for, there remained no significant association between plasma IL-1Ra and indices of insulin secretion (Log_10_ second-phase insulin, total insulin and C-peptide). Thus, as with plasma apoB, the association of IL-1Ra with insulin secretion is not independent of IR.

### The association of hyperapoB to hyperinsulinemia and IR is dependent on plasma IL-1Ra

We examined whether the relation of plasma apoB to hyperinsulinemia and IR was dependent on the IL-1 family by two methods. First, in a partial correlation analysis, adjusting for plasma IL-1Ra eliminated the association of plasma apoB with all indices of insulin secretion and sensitivity (*P*>0.05 for all indices in Figure 3). Adjusting this model for IL-1β had no effect. Second, we divided the study population into quintiles and compared subjects with hyperapoB (>80th percentile of plasma apoB) and those with low plasma apoB (<20th percentile). Given the sex differences in insulin sensitivity and secretion described above, we assured equal number of men and women in each group by selecting the quintiles per sex. This resulted in 7 men and 10 women in each group. Of note, plasma apoB in the low apoB and hyperapoB groups corresponded to <25th percentile (apoB=0.79 g l^−1^) and ⩾75th percentile (apoB=1.17 g l^−1^) of a larger Canadian population (*N*=3519), respectively.^43^

Although no differences existed in any index of adiposity or body fat distribution, subjects with hyperapoB had higher indices of insulin secretion (fasting and total C-peptide secretion), IR (lower GIR and M/I) and elevated plasma IL-1Ra (Table 2 and Supplementary Figure S2). Adjustment for obesity indices (BMI, total, android, gynoid, or android/gynoid fat ratio, waist or waist/hip ratio) or for sex did not eliminate group differences in indices of insulin sensitivity, C-peptide secretion and plasma IL-1Ra. However, adjusting for IL-1Ra alone, but not IL-1β, eliminated all group differences in insulin sensitivity and secretion.

## Discussion

Here we present in a population of 81 overweight and obese yet normoglycemic subjects that (1) independent of sex, obesity and body composition, plasma apoB was positively associated with hyperinsulinemia, IR and plasma IL-1Ra and (2) the association of plasma apoB to hyperinsulinemia and IR was eliminated when adjusted for plasma IL-1Ra suggesting the involvement of an activated IL-1 family.

Before embarking on data interpretation, three important points need to be underscored. First, the correlative nature of this study does not imply causality; however, it provides a translation of the basic findings on the relation of apoB-lipoproteins to the activation of the inflammasome^31, 32^ into clinical observations. Moreover, it allows for the generation of novel hypotheses regarding the link between atherogenic lipoproteins and risk factors for T2D in obese subjects. This is particularly strengthened by the use of two gold-standard tests to independently measure insulin secretion and sensitivity, which reduces the risk of auto-correlation that may be generated using a single test.^44^

Second, the associations of plasma apoB and IL-1 family with insulin sensitivity and secretion should be evaluated in the context of the pathophysiology of T2D and the health status of the population examined. Current evidence on the progression of normal glucose tolerance to T2D in humans favors a two-step model. In the first step, normal glucose progresses to impaired glucose tolerance, with IR driving higher insulin secretion.^45, 46^ In the second step, impaired glucose tolerance progresses to T2D, with the progressive loss of β-function and eventual decline in insulin secretion. Secretion of insulin encompasses two phase: the first phase involves the fusion of a small 'readily releasable pool' of granules (~50–200) that are predocked,^47^ or situated close to,^48^ the plasma membrane leading to the quick discharge of insulin and is the first to decline during the progressive loss of β-function.^49^ The second phase represents a 'reserve pool' of storage granules that are mobilized in response to glucose and produces a substantial and prolonged insulin secretion.^47^ Quantitatively, the second-phase insulin secretion is larger (~80%)^47^ as also demonstrated in this study. Despite their obesity and age range that increase their risk to T2D, subjects examined this study were free of chronic disease and with normal fasting glucose on average. They represented an early stage in step 1 toward progression to T2D, if ever. Therefore, it is not surprising that there was no associations of plasma apoB and IL-1 family with first-phase insulin secretion and that their association with second phase and IR were not too strong. Stronger associations may be observed with the progression of prediabetic to diabetic states; however, this remains to be explored in future studies.

Third, men had higher IR and secretion than women. Similar sex differences in insulin sensitivity were observed by some studies using the hyperinsulinemia clamp^50, 51^ and frequently sampled IVGTT^52^ but not by insulin-suppression test.^52^ Men were also found to have lower whole-body insulin clearance,^53^ which may explain their higher plasma insulin during the Botnia clamp. These findings underscore the need to adjust for plasma insulin when insulin sensitivity is examined in both sexes during the hyperinsulinemia clamp, as is carried out in this study using M/I.

Nevertheless, despite these sex differences, the association of plasma apoB with IR and hyperinsulinemia was independent of sex or body composition and may be related to the effects of apoB-lipoproteins *per se*. Elevated concentrations and uptake of apoB-lipoproteins into peripheral tissue promote multiple degenerations. In our hand, differentiation of 3T3-L1 preadipocytes with elevated but physiological concentrations of LDL (1.4 g l^−1^ apoB) reduced adipocyte function assessed as the hydrolysis and clearance of triglyceride-rich lipoproteins.^7^ In line, postmenopausal obese women with high plasma apoB had delayed postprandial plasma fat clearance *in vivo* and dysfunctional white adipose tissue *ex vivo*.^7^ Similarly, oxidized LDL were reported to increase 3T3-L1 proliferation and decrease differentiation,^8^ a defect that was dependent on scavenger receptor, CD36.^54, 55^ In the muscle, TRL remnants^9^ and ceramide-rich LDL^13^ inhibited insulin action in rat L6 muscle cells, which was reversed by inhibition of LDL receptor family.^9^ Finally, human β-cells express LDL receptors^56^ and internalization of LDL by β-cell induce β-cell dysfunction and apoptosis.^10, 11, 12^ It should be noted, however, that while apoB-lipoproteins induce multiple degenerations in β-cells, their association with insulin secretion *in vivo* was likely driven by their effects in insulin-sensitive tissues, as adjusting for insulin sensitivity in this study eliminated the association of apoB with glucose-induced C-peptide secretion.

Finally, adjusting the association of plasma apoB with IR and hyperinsulinemia for plasma IL-1Ra eliminated this association, suggesting the involvement of the IL-1 family *in vivo*. *In vitro*, IL-1β has been shown to reduce adipose tissue ability to hydrolyze triglycerides^57^ and to interfere with adipocyte differentiation.^58^ Moreover, while low levels of IL-1β are important for β-cell function,^59^ excess IL-1β is implicated in β-cell deterioration and the development of T2D,^20, 60^ and IL-1β is overexpressed in the islets of T2D patients.^61^ This is in line with the positive association of plasma IL-1β with glucose-induced insulin secretion in our population, despite that plasma IL-1β was near detectable limits. Notably, administration of Anakinra, a recombinant IL-1Ra, improved first-phase insulin secretion in subjects with impaired glucose tolerance and insulin production and glycemic control in patients with T2D,^21, 62^ findings which were confirmed using anti-IL-1β in T2D patients.^21^ This supports the involvement of IL-1, particularly IL-1β, in the pathogenesis of T2D. Of note, while it cannot be excluded that the association of plasma apoB with hyperinsulinemia and IR may also be related to increased production of IL-1α, which promotes IL-1Ra secretion, IL-1α is rarely found in circulation and its role in T2D in humans is less clear.^20, 21^

IL-1β is the end product of the activation of the NLRP3 inflammasome, which is implicated in the recognition of obesity-associated metabolic signals in macrophage and pancreatic cells.^19, 20^ ApoB-lipoproteins, very LDL^31^ and oxidized LDL^32^ activate the NLRP3 inflammasome leading to the expression and secretion of IL-1β from monocytes and macrophages. Thus it is plausible that apoB-lipoproteins may be recognized by the NLRP3 in insulin-sensitive tissues such as adipose tissue and muscle and by the pancreas. This in turn may activate the inflammasome, leading to local hypersecretion of IL-1β compared with IL-1Ra, which is reflected *in vivo* by elevated IL-1Ra, and progression of IR to T2D. We hypothesize that reducing circulating levels of apoB-lipoproteins, without increasing their uptake into peripheral tissues, may reduce the activation of the NLRP3 inflammasome and the risks for T2D.

In conclusion, elevated plasma apoB associates with hyperinsulinemia and IR independent of adiposity in normoglycemic overweight and obese subjects. This association may be dependent on the activation of the IL-1 family, particularly IL-1β and IL-1Ra.

## Figures and Tables

**Table 1:** Fasting baseline anthropometric and metabolic characteristics and indices of insulin sensitivity and glucose-induced insulin secretion during the Botnia clamp in the study population

*Characteristics*	*Women (*N=*48)*	*Men (*N=*33)*	P-*value*
Age (years)	58.2±0.82	56.6±1.16	0.250
Weight (kg)	81.6±1.8	103.6±3.6	<0.001
BMI (kg m^−2^)	32.2±0.59	34.0±0.97	0.093
Total fat mass (kg)	37.4±1.2	38.4±2.5	0.689
Lean body mass (kg)	41.3±0.7	61.5±1.4	<0.001
Android fat mass (kg)	3.48±1.47	4.59±3.15	0.001
Gynoid fat mass (kg)	6.39±0.20	5.14±0.35	<0.001
Android/gynoid	0.55±0.02	0.91±0.03	<0.001
Waist circumference (cm)	102.3±1.5	116.3±2.5	<0.001
Hip circumference (cm)	112±2	113±1	0.535
Waist/hip ratio	0.90±0.01	1.04±0.01	<0.001
Plasma apoB (g l^−1^)	0.95±0.04	1.00±0.04	0.374
Plasma total cholesterol (mmol l^−1^)	5.65±0.14	4.99±0.16	0.003
Plasma non-HDL cholesterol (mmol l^−1^)	4.13±0.16	3.95±0.17	0.458
Plasma LDL cholesterol (mmol l^−1^)	3.40±0.12	2.88±0.11	0.004
Plasma HDL cholesterol (mmol l^−1^)	1.53±0.06	1.04±0.04	<0.001
Mean LDL size (A°)	268.7±0.7	265.4±1.1	0.013
Plasma TG (mmol l^−1^)	1.61±0.14	2.36±0.29	0.011
Plasma NEFA (mmol l^−1^)	0.507±0.024	0.408±0.022	0.005
Plasma ApoA-I (g l^−1^)	1.68±0.04	1.40±0.03	<0.001
Systolic blood pressure (mm Hg)	120±2	130±2	0.005
Diastolic blood pressure (mm Hg)	75±1	82±1	<0.001
Plasma IL-1β (pg ml^−1^)	0.101±0.004	0.099±0.004	0.729
Plasma IL-1Ra (pg ml^−1^)	336±46	334±35	0.976
Fasting plasma glucose (mmol l^−1^)	5.12±0.07	5.31±0.08	0.102
Fasting plasma insulin (μU ml^−1^)	21.4±4.5	28.76±4.14	0.257
Fasting plasma C-peptide (ng ml^−1^)	2.0±0.1	2.7±0.2	0.001
Fasting HbA_1_C	0.057±0.001	0.056±0.001	0.057
Fasting QUICKI	0.315±0.003	0.301±0.003	0.002
First-phase insulin secretion_IVGTT_ (μU ml^−1^)	769.0±69.0	1135.7±157.0	0.020
Second-phase insulin secretion_IVGTT_ (μU ml^−1^)	2623.9±269.5	4639.5±620.5	0.001
Total insulin secretion_IVGTT_ (μU ml^−1^)	3466±373	5775±749	0.004
AUC_IVGTT_ glucose (mmol l^−1^)	655±13	677±13	0.249
Total C-peptide secretion_IVGTT_ (ng ml^−1^)	287.7±16.2	371.8±29.2	0.009
Insulin at steady state_Clamp_ (μU ml^−1^)	255.9±10.4^a^	294.0±18.3^b^	0.058
Glucose infusion rate_Clamp_ (mg (kg × min)^−1^)	12.6±0.6^a^	9.3±0.6	<0.001
M/I_Clamp_ (mg (kg × min)^−1^)/(μU ml^−1^)	0.054±0.004^a^	0.036±0.004^b^	0.002
DI_First__-__phase insulin secretion_ (mg (kg × min)^−1^)	35.3±3.2	35.4±4.2	0.989
DI_Total insulin secretion_ (mg (kg × min)^−1^)	147.8±9.1	162.8±12.6	0.325

Abbreviations: AUC, area under the curve; BMI, body mass index; DI, disposition index; HbA_1_C, glycated hemoglobin; HDL, high-density lipoproteins; IL, interleukin; IVGTT, intravenous glucose tolerance test; LDL, low-density lipoproteins; M/I, glucose infusion rate/steady-state plasma insulin; NEFA, non-essential fatty acid; QUICKI, quantitative insulin sensititvity check index; TG, triglycerides. Data are presented as mean±s.e.m.

a *N*=44 missing samples.

b *N*=32 missing samples.

**Table 2:** Characteristics of subjects with low and high plasma apoB

*Characteristics*	*Low plasma apoB (<20th* *percentile,* N=*17)*	*HyperapoB (>80th* *percentile,* N=*17)*
Plasma apoB (g l^−1^)	0.66±0.03	1.32±0.04***
(min–max)	0.34–0.78	1.14–1.80
Sex (male:female)	7:10	7:10
Age (years)	56.2±1.1	59.4±1.3
Weight (kg)	91.4±5.3	89.6±4.4
BMI (kg m^−2^)	33.8±1.2	32.9±1.2
Total fat mass (kg)	38.4±2.8	37.7±2.6
Lean body fat (kg)	49.4±3.1	48.7±2.6
Android fat (kg)	3.98±0.43	4.11±0.34
Gynoid fat (kg)	6.09±0.44	5.69±0.39
Android/gynoid	0.676±0.059	0.744±0.059
Waist circumference (cm)	111±4	108±3
Waist/hip circumference	0.98±0.03	0.97±0.02
Plasma total cholesterol (mmol l^−1^)	4.42±0.16	6.57±0.23***
Plasma non-HDL cholesterol (mmol l^−1^)	2.91±0.14	5.33±0.23***
Plasma LDL cholesterol (mmol l^−1^)	2.39±0.13	4.02±0.16***
Plasma HDL cholesterol (mmol l^−1^)	1.51±0.15	1.24±0.09
Mean LDL size (A°)	270±1	264±2**
Plasma TG (mmol l^−1^)	1.14±0.17	2.87±0.49***
Plasma NEFA (mmol l^−1^)	0.474±0.047	0.498±0.031
Plasma ApoA-I (g l^−1^)	1.58±0.08	1.59±0.07
Systolic blood pressure (mm Hg)	123±5	127±4
Diastolic blood pressure (mm Hg)	76±3	79±1
Plasma IL-1β (pg ml^−1^)	0.094±0.004	0.091±0.002
Plasma IL-1Ra (pg ml^−1^)	269±34	463±113^a^*
Fasting plasma glucose (mmol l^−1^)	5.10±0.09	5.36±0.17
HbA1c	0.056±0.001	0.058±0.001^b^
Fasting plasma insulin (μU ml^−1^)	19.4±1.8	22.8±2.3
Fasting plasma C-peptide (ng ml^−1^)	2.15±0.21	2.77±0.22*
Fasting QUICKI	0.312±0.005	0.303±0.004
First-phase insulin secretion_IVGTT_ (μU ml^−1^)	948±180	1087±136
Second-phase insulin secretion_IVGTT_ (μU ml^−1^)	2898±375	4052±611
Total insulin secretion_IVGTT_ (μU ml^−1^)	3646±509	5138±724
Total C-peptide secretion_IVGTT_ (ng ml^−1^)	291±33	394±38*
Insulin at steady state_clamp_ (μU ml^−1^)	255±13	276±21
Glucose infusion rate_Clamp_ (mg (kg × min)^−1^)	12.2±1.0	9.1±1.1*
M/I_Clamp_ (mg (kg × min)^−1^)/(μU ml^−1^)	0.052±0.007	0.037±0.006*
Disposition index (mg (kg × min)^−1^)	163±15	159±20

Abbreviations: BMI, body mass index; HbA_1_C, glycated hemoglobin; HDL, high-density lipoproteins; IL, interleukin; IVGTT, intravenous glucose tolerance test; LDL, low-density lipoproteins; M/I, glucose infusion rate/steady-state plasma insulin; NEFA, non-essential fatty acid; QUICKI, quantitative insulin sensititvity check index; TG, triglycerides. Data are presented as mean±s.e.m. *P⩽0.05 and **P⩽0.01, ***P⩽0.001 for group differences by unpaired *t*-test. Statistical analysis was conducted on the Log_10_ of insulin and C-peptide secretions, M/I and IL-1Ra.

a *N*=16 missing samples.

b *N*=15 missing samples.
