# Supplementary material for: Plasma IL-1Ra: linking hyperapoB to risk factors for type 2 diabetes independent of obesity in humans
Source: Nutr Diabetes. 2015 Sep 28;5(9):e180–. doi: 10.1038/nutd.2015.30 (PMC4657760; doi:10.1038/nutd.2015.30)
Supplement: Supplementary Information [file nutd201530x1.docx]

**Supplement Table 1:** Association of anthropometric and metabolic parameters with insulin secretion and sensitivity and plasma IL-1β and IL-Ra in 81 overweight and obese subjects (N=48 women and N=33 men)

| Parameters | QUICKI | Log_10_  [M/I] | | Log_10_  [1^st^ phase insulin secretion] | | Log_10_  [2^nd^ phase insulin secretion] | | Log_10_  [Total insulin secretion] | Log_10_  [Total plasma C-peptide] | | Log_10_  [Plasma IL-1β] | | Log_10_  [Plasma  IL-1Ra] | | Log_10_  [IL-1Ra/  IL-1β] | |
| --- | --- | --- | --- | --- | --- | --- | --- | --- | --- | --- | --- | --- | --- | --- | --- | --- |
| *Anthropometric parameters* | | |  | |  | |  | | |  | |  | |  | |  |
| Weight | **-0.39**** | **-0.50**** | | **0.32**** | | **0.51**** | | **0.48**** | **0.47**** | | -0.04 | | **0.39**** | | **0.31**** | |
| BMI | **-0.36**** | **-0.48**** | | **0.27*** | | **0.46**** | | **0.42**** | **0.44**** | | 0.04 | | **0.45**** | | **0.31**** | |
| Total fat | **-0.28**** | **-0.44**** | | **0.25*** | | **0.39**** | | **0.37**** | **0.47**** | | 0.05 | | **0.50**** | | **0.34**** | |
| Lean body mass | **-0.37**** | **-0.48**** | | **0.26*** | | **0.45**** | | **0.42**** | **0.31**** | | -0.10 | | 0.16 | | 0.19 | |
| Android fat | **-0.40**** | **-0.52**** | | **0.31**** | | **0.52**** | | **0.48**** | **0.53**** | | 0.04 | | **0.54**** | | **0.37**** | |
| Gynoid fat | -0.04 | **-0.23*** | | 0.15 | | 0.12 | | 0.13 | **0.27*** | | 0.08 | | **0.28**** | | 0.15 | |
| Android/ gynoid | **-0.36**** | **-0.32**** | | 0.14 | | **0.40**** | | **0.35**** | **0.29**** | | -0.06 | | **0.29**** | | **0.26*** | |
| Waist | **-0.46**** | **-0.48**** | | **0.33**** | | **0.52**** | | **0.52**** | **0.51**** | | 0.03 | | **0.41**** | | **0.29*** | |
| Hip | -0.18 | **-0.37**** | | 0.15 | | **0.25*** | | **0.24*** | **0.35**** | | 0.13 | | **0.37**** | | 0.18 | |
| Waist/Hip ratio | **-0.43**** | **-0.44**** | | **0.28*** | | **0.47**** | | **0.44**** | **0.35**** | | 0.09 | | 0.20 | | 0.20 | |
| *Fasting lipoproteins parameters* | | | | |  | |  | | |  | |  | |  | |  |
| ApoB | -0.16 | **-0.29*** | | 0.13 | | **0.24*** | | **0.22*** | **0.26*** | | -0.09 | | **0.26*** | | **0.25*** | |
| ApoA1 | 0.20 | **0.34**** | | -0.27* | | **-0.33**** | | **-0.33**** | **-0.34**** | | -0.01 | | **-0.11** | | -0.08 | |
| Total cholesterol | -0.04 | -0.03 | | 0.04 | | 0.07 | | 0.07 | 0.15 | | -0.03 | | 0.14 | | 0.12 | |
| Non HDL-C | -0.16 | -0.17 | | 0.12 | | 0.21 | | 0.20 | **0.26*** | | -0.05 | | 0.21 | | 0.19 | |
| LDL-C | -0.04 | -0.04 | | 0.04 | | 0.07 | | 0.06 | 0.14 | | -0.01 | | **0.22*** | | 0.17 | |
| HDL-C | **0.30**** | **0.33**** | | -0.20 | | **-0.35**** | | **-0.33**** | **-0.28*** | | 0.06 | | -0.19 | | -0.19 | |
| TG | **-0.22*** | **-0.24*** | | 0.16 | | **0.27*** | | **0.25*** | **0.27*** | | -0.08 | | 0.07 | | 0.10 | |
| NEFA | -0.09 | -0.12 | | -0.06 | | -0.07 | | -0.08 | 0.04 | | 0.16 | | **0.30**** | | 0.11 | |
| Mean LDL size | **0.23*** | **0.22** | | -0.21 | | **-0.29**** | | **-0.28*** | **-0.34**** | | -0.06 | | -0.11 | | -0.04 | |

* for *p* ≤ 0.05 and ** for *p* ≤ 0.01 by Pearson correlation. Units of presented parameters are equivalent to those in Table I.

**Supplement Figure 1:** Correlation of plasma apoB with BMI (A) weight (B) waist circumference (C), hip circumference (D), total fat mass (E), android fat mass (F), gynoid fat mass (G), android/gynoid fat mass (H) in women (N=48, open circles) and men (N=33, solid circles).

**Supplement Figure 2 :** Differences in subjects with low plasma apoB versus hyperapoB in plasma IL-1Ra (A) total C-peptide secretion (B), insulin sensitivity as M/I (C), insulin sensitivity as GIR (D) in women (N=10, open circles) and men (N=7, solid circles). Data is presented as scatter plot with pooled mean in each group.

**Supplement Figure 1**

**Supplement Figure 2**
